# Luminal plant sterol promotes brush border membrane-to-lumen cholesterol efflux in the small intestine

**DOI:** 10.3164/jcbn.17-116

**Published:** 2018-03-30

**Authors:** Takanari Nakano, Ikuo Inoue, Yasuhiro Takenaka, Yuichi Ikegami, Norihiro Kotani, Akira Shimada, Mitsuhiko Noda, Takayuki Murakoshi

**Affiliations:** 1 Department of Biochemistry, Faculty of Medicine, Saitama Medical University, Iruma, Saitama 350-0495, Japan; 2 Department of Diabetes and Endocrinology, Faculty of Medicine, Saitama Medical University, Iruma, Saitama 350-0495, Japan; 3 Department of Physiology, Nippon Medical School, Bunkyo-ku, Tokyo 113-8602, Japan

**Keywords:** cholesterol absorption, phytosterols, trans-intestinal cholesterol efflux, small intestine

## Abstract

Plant sterols are used as food additives to reduce intestinal cholesterol absorption. They also increase fecal neutral sterol (FNS) excretion irrespective of the absorption inhibition. Intestine-mediated reverse cholesterol transport, or trans-intestinal cholesterol efflux (TICE), provides the major part of the increase of FNS excretion. However, it is unknown whether plant sterols stimulate TICE or not. We have shown previously that TICE can be evaluated by brush border membrane (BBM)-to-lumen cholesterol efflux. Thus, we examined whether luminal plant sterols stimulate BBM-to-lumen cholesterol efflux in the intestinal tract or not in mice. Cannulated upper jejunum that had been pre-labeled with orally given ^3^H-cholesterol, was flushed and perfused to collect ^3^H-cholesterol effluxed back into the lumen from the BBM to estimate the efflux efficiency. Adding 0.5 mg/ml of plant sterols, but not cholesterol, in the perfusion solution doubled the efflux. Plant sterols enter the BBM and are effluxed back to the lumen rapidly, in which process cholesterol transporters in the BBM are involved. We thus speculate that phytosterols alter cholesterol flux in the BBM; thereby, increases BBM-to-lumen cholesterol efflux, resulting in the increased TICE.

## Introduction

Circulating cholesterol accumulates in the artery walls slowly, but steadily, over the life span. To keep normocholesterolemia helps prevent from such deposition, or atherosclerosis. Most of the cholesterol in the body should ultimately be excreted as bile acid or as it is to feces to maintain endogenous cholesterol balance. The hepato-bilial reverse cholesterol transport is known to mediate cholesterol disposal from the peripheral tissues into the intestinal lumen. On the other hand, emerging evidence has shown that the small intestine also excretes large amounts of endogenous cholesterol into the intestinal lumen, a phenomenon designated as trans-intestinal cholesterol efflux (TICE).^(1)^

A typical diet contains non-cholesterol sterol analogs, such as plant sterols and plant stanols, which are referred to as ‘phytosterols’ hereafter, in a similar amount to cholesterol. Phytosterols are used as food additives to lower plasma cholesterol level, because ingesting such non-cholesterol sterols in excess reduces intestinal cholesterol absorption.^(2)^ Surprisingly, such intake also stimulates FNS excretion approximately by 4-fold in mice. TICE is probably a major source for the increase, because the intake does not alter the rate of biliary cholesterol secretion,^(3)^ indicating that the increase originates an extra-hepatic action(s). Also, cholesterol (re)absorption inhibition could account for the increase only in a part with a careful calculation.^(3)^

In a previous study, we developed a method that determines brush border membrane (BBM)-to-lumen cholesterol efflux.^(4)^ We found that the increased efflux estimated the increase of TICE. Indeed, ezetimibe, a potent TICE and FNS excretion inducer,^(5)^ increased the efflux by 20-fold at maximum.^(4)^ In the present study, we attempted to examine whether luminal phytosterols stimulate BBM-to-lumen cholesterol efflux as a possible mechanism for the promotion of TICE by phytosterol intake.

## Materials and Methods

### Luminal perfusion assay

BBM-to-lumen cholesterol efflux was determined by a luminal perfusion assay in mice.^(4)^ Briefly, eight-to-ten-week old male C57BL/6J mice were given 5 µCi of ^3^H-cholesterol by gavage with 100 µl triolein to label the intestinal epithelia with the given tracer. Three h after infusion, we opened the abdomen of anesthetized mice, cannulated a portion of the upper small intestinal segment (approx. 5 cm), and flushed the luminal content with Krebs-HEPES buffer from the proximal to the distal end at a flow rate of 0.25 ml/min for 12 min. The segments were then perfused with the same buffer containing taurocholic acid and phosphatidylcholine at a flow rate of 0.05 ml/min for 1 h. To mimic phytosterol-mediated cholesterol efflux, we added phytosterols [0.5 mg/ml; a phytosterol cocktail, Thermo Fisher Scientific Inc., Waltham, MA, cat. no. 13272, Lot. A0265923; β-sitosterol, 78.2%; β-sitostanol, 10.6%; campesterol, 7.5%; campestanol, 0.9% (w/w)] to the perfusion buffer. Perfusate aliquots were collected for 15 min each, and designated as fractions #1, #2, #3, and #4. Each fraction was counted for ^3^H radioactivity. We calculated the efflux efficiency (%) using the following formula:

 

Tracer count in the perfusate (decay per minute, DPM)/tracer count in intestinal segment perfused (DPM) × 100

 

All animal experiments were approved by the Animal Care Committee of Saitama Medical University.

### Circulation-to-lumen sterol transit assay in mice

 Circulation-to-lumen sterol transit assays were performed as previously described,^(4)^ originally developed by van der Velde *et al.*^(1)^ Briefly, we cannulated the right jugular vein and ligated the common bile duct and the small intestinal segments (approx. 5 cm) of anesthetized C57BL/6J mice. One hundred µl Intralipid [20% (w/v) emulsion] containing either of 10 µCi ^3^H-cholesterol, ^3^H-sitosterol, or ^3^H-sitostanol tracer was injected into the jugular vein, and the cannulated intestinal segment was perfused as above. The perfused aliquots were collected for 10 min each (1 h in total). After the perfusion, blood was collected, and the cannulated intestinal segments were excised after the remaining blood in the circulation was flushed out with saline. We estimated TICE as follows:

Tracer count in the perfusates (DPM)/length of the small intestine perfused (cm)/tracer count of serum samples (100 µl, DPM).

### Serosal-to-lumen sterol transit assay in Caco-2 cells

Differentiated Caco-2 cell monolayers on the permeable cell culture insert were prepared as described previously.^(4)^ Briefly, DMEM containing lipid micelles and 1 µCi/ml ^3^H-cholesterol was added to differentiated Caco-2 cells from the basolateral side. The cell were incubated at indicated periods, then the cells, the apical and basolateral media were counted for ^3^H tracer activity.

## Results and Discussion

**Fig. 1:**
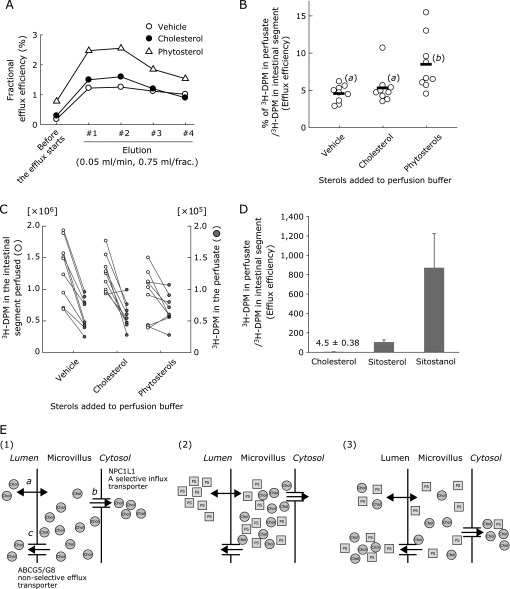
Luminal phytosterols increase intestinal cholesterol efflux. (A) Elution patterns of ^3^H-cholesterol from the cannulated intestinal segments. Each plot indicates the mean (*n* = 9). (B) Efflux efficiency (%; sum of #1–#4) in the presence of phytosterols or cholesterol in the luminal perfusate. Different letters in parenthesis indicate a statistically significant difference between the groups using Tukey’s Honestly Significant Difference test. (C) Plots for ^3^H-decay per minute (DPM) in the perfusate (left; sum of #1–#4) and intestinal segment perfused (right). (D) Sterol efflux efficiency determined by luminal perfusion assay (the original data were previously reported and are shown as a liner bar graph with mean ± SD).^(3)^ (E) Proposed model for phytosterol-mediated brush border membrane (BBM)-to-lumen cholesterol efflux. (1) a, sterols diffuse into or out of the BBM; b, Niemann-Pick C1-like 1 (NPC1L1), a sterol-selective influx transporter, transfers sterols, preferentially cholesterol, for further absorptive processes. c, ATP-binding cassette (ABC) G5/G8 pumps out sterols to the lumen non-selectively. (2) and (3) show phytosterols (PS) behavior when present in the intestinal lumen in a successive manner (2 to 3). (2), Luminal phytosterols enter the BBM and compete with cholesterol already present in the BBM. (3), When sterols in the BBM are effluxed into the lumen, both the passive diffusion and ABCG5/G8 do not discriminate between sterol species and, as a result, cholesterol present in the BBM is diluted with plant sterols. Such dilution of cholesterol in the BBM can reduce the efficiency of NPC1L1-mediated cholesterol transport.

Ingestion of phytosterols increased FNS excretion in mice probably via TICE,^(3)^ but direct and supportive evidence for it has been lacking. In the present study, luminal perfusion assays showed that the addition of phytosterols in the perfusion solution increased BBM-to-lumen efflux of ^3^H-cholesterol incorporated in the cannulated segments of the small intestine over the perfusion period (Fig. 1A). Area under the curve analysis showed that the addition of phytosterols doubled the efflux (Fig. 1B). Such an effect was not observed when cholesterol was added to the perfusion solution instead. The addition of such sterols to the perfusate did not alter ^3^H-cholesterol tracer abundance in the intestinal segments (Fig. 1C), excluding analytical bias when calculating the efflux ratios.

We and others found that phytosterols are taken up by the BBM in the small intestine similar to cholesterol,^(4,6)^ followed by rapid efflux back to the lumen compared with cholesterol (Fig. 1D),^(4)^ preventing the further absorption of the harmful substances.^(7)^ ATP-binding cassette (ABC) G5/G8 sterol transporter complex, which is localized at the BBM, mediates the efflux in a part.^(4,5)^ This dimeric transporter pumps out both cholesterol and non-cholesterol sterols and the BBM is the major site of cholesterol storage in the cells. We thus speculated that cholesterol in the BBM is concomitantly effluxed to the lumen when luminal phytosterol enters the BBM and exits it through the transporter (Fig. 1E).

There are a couple of other substances that have shown to stimulate TICE so far. Ezetimibe inhibits Niemann-Pick C1-like 1, a BBM-localized sterol transporter, increases TICE.^(5,8)^ A farnesoid X receptor agonist PX20606 also stimulates TICE via an ABCG5/G8-dependent manner. Live-X-receptor agonists, which stimulates cholesterol efflux by modulating the expression of the genes related to BBM-localized sterol transport such as ABCG5/G8 sterol transporter complex, also increase FNS excretion probably as a result of TICE stimulation.^(9,10)^ These suggest that the BBM is a major site that is associated with the modulation of TICE.

**Fig. 2:**
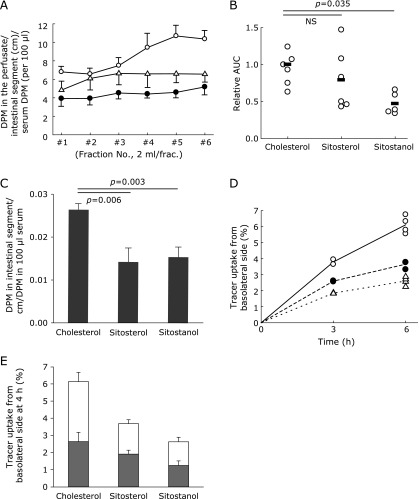
Subapical components of the enterocytes do not accelerate phytosterol efflux. (A) Circulation-to-lumen sterol efflux in mice. Open circles, cholesterol (*n* = 9); Closed circles, sitosterol (*n* = 9); Open triangles, sitostanol (*n* = 7). (B) Comparison of the area under the curves in A. The data were compared by the Dunnett’s test with cholesterol as the reference. (C) ^3^H-tracer count (DPM) in the intestinal segments normalized by the length of the intestinal segment perfused and the ^3^H-tracer count (DPM) in the blood. (D) ^3^H-sterol tracer uptake from the basolateral side of the Caco-2 cell monolayers. Open circles, cholesterol; Closed circles, sitosterol; Open triangles, sitostanol. *n* = 4 of each group. (E) Distribution of sterol tracer at 4 h incubation. Gray bars, tracer count in the cells; open bars, tracer count in the apical media at 4 h incubation. Bars indicate mean ± SD.

To exclude the involvement of the subapical components in the cytosol and the basolateral membranes in the phytosterol-mediated TICE, we estimated how rapidly phytosterol moves during the transit from the circulation to the small intestinal lumen in comparison with cholesterol. In mice, when sitosterol or sitostanol tracer was infused into the circulation, sitostanol transit to the lumen was slower than cholesterol (Fig. 2A and B) and the transits both phytosterols to the intestinal segments were as well (Fig. 2C). In Caco-2 cells, serosal-to-lumen transits of the two phytosterols tested were approximately a half than that of cholesterol (Fig. 2D and E). Cells-to-lumen transit of the tracers (open bars vs gray bars) showed no apparent differences between cholesterol and phytosterols (Fig. 2E). These results indicate that the intracellular and basolateral sterol transport machineries are not involved in the rapid phytosterol efflux, supporting that the event occurs within the BBM (Fig. 1E).

The results of the present study support the idea the phytosterol-mediated FNS increase originates from the small intestine and that TICE plays a major role. Although phytosterols have not been reported to have any molecular targets in the BBM, the presence in the intestinal lumen affects the cholesterol flux (Fig. 1). We speculated an increase of concomitant efflux of cholesterol occurs when abundant phytosterol enters the BBM, but further evidence, such as experiments with vesicle models or artificial membranes for example, should be needed to conclude it.

Phytosterol intake is considered to be safe because little is absorbed because of the elimination at the gut;^(11)^ however, there has still been a dispute.^(12)^ Understanding the efficacy and mechanism would help further development and utilization of phytosterols and our findings may shed light on further benefit of phytosterols for clinical use.

## Abbreviations

BBM: brush border membrane
DPM: decay per minute
TICE: trans-intestinal cholesterol efflux
